# Pyroptosis-immunity-microbiome axis in acute upper gastrointestinal bleeding: mechanisms, risk prediction, and individualized strategies

**DOI:** 10.3389/fmed.2026.1780566

**Published:** 2026-06-01

**Authors:** Lijun Mei, Piao Zhang, Tao Xiang, Chengcheng Sun

**Affiliations:** 1 Department of Health Management Center, The Third People’s Hospial of Chengdu, The Affliated Hospital of Southwest Jiao Tong University, Chengdu, Sichuan, China; 2 Department of Anaesthesiology, West China Hospital, Sichuan University, Chengdu, Sichuan, China; 3 Department of Emergency Medicine, Chengdu Third People’s Hospital, Chengdu, Sichuan, China

**Keywords:** immunity, microbiome, pyroptosis, risk prediction, upper gastrointestinal bleeding

## Abstract

Acute upper gastrointestinal bleeding (UGIB) is a critical emergency commonly encountered in gastroenterology. Its pathogenesis is complex and involves diverse etiologies. Emerging evidence indicates that pyroptosis, dysregulated immune-inflammatory responses, and gut microbiome imbalance are pivotal mechanisms driving gastric mucosal injury and hemorrhage. This review systematically synthesizes the risk factors, pathophysiological mechanisms, risk prediction models, and therapeutic strategies for UGIB, with particular emphasis on the intricate interplay among pyroptosis, immunity, and the microbiome and on their value as potential therapeutic targets. We first summarize the common etiologies and risk factors of UGIB, including pharmacological agents, infections, advanced age, comorbidities, and genetic predispositions. We then delineate the pathogenic role of pyroptosis in gastric mucosal injury, with particular focus on activation of the GKN2-NLRP3 axis. Next, we discuss the utility of systemic inflammatory markers such as the neutrophil-to-lymphocyte ratio (NLR) and C-reactive protein (CRP) in UGIB risk stratification, together with the mechanisms by which gut microbiome dysbiosis compromises mucosal barrier integrity and amplifies inflammatory responses through microbial metabolites and pathogen translocation. The core section provides an in-depth analysis of the reciprocal, self-amplifying network linking pyroptosis, immune activation, and microbiome perturbation, thereby elucidating the basis for the frequent co-occurrence of systemic inflammation and microbial dysbiosis in UGIB. Finally, we critically evaluate established risk-scoring systems (Glasgow-Blatchford Score, Rockall score, and AIMS65) and emerging biomarkers. Overall, this review assesses emerging therapeutic strategies, including pyroptosis inhibitors and microbiome-modulating interventions, and provides a theoretical framework for personalized management of UGIB.

## 1 Introduction

Acute upper gastrointestinal bleeding (UGIB) constitutes a major public health concern globally, with an annual incidence of approximately 48 to 160 per 100,000 population and in-hospital mortality rates ranging from 5 to 15% (1). The clinical manifestations of UGIB include hematemesis, melena, hypotension, and shock, and adverse outcomes are closely associated with bleeding etiology, blood loss volume, patient age, and comorbidities (1, 2). Conventional research has predominantly focused on mechanical trauma and acid-mediated mucosal injury. However, emerging evidence suggests that inflammatory programmed cell death (pyroptosis), immune dysregulation, and gut microbiome dysbiosis collectively orchestrate a multistep pathogenic cascade involving mucosal injury, vascular fragility, and coagulopathy (3). Concurrently, systemic inflammatory markers—specifically the neutrophil-to-lymphocyte ratio (NLR) and C-reactive protein (CRP)—together with validated risk scoring systems (Glasgow-Blatchford, Rockall, and AIMS65), are widely used for UGIB risk stratification (1, 2). Nevertheless, current understanding of the pyroptosis-immune-microbiome interplay remains limited, and pyroptosis-targeted therapeutics alongside individualized prognostic models are not yet clinically mature.

**Figure 1:**
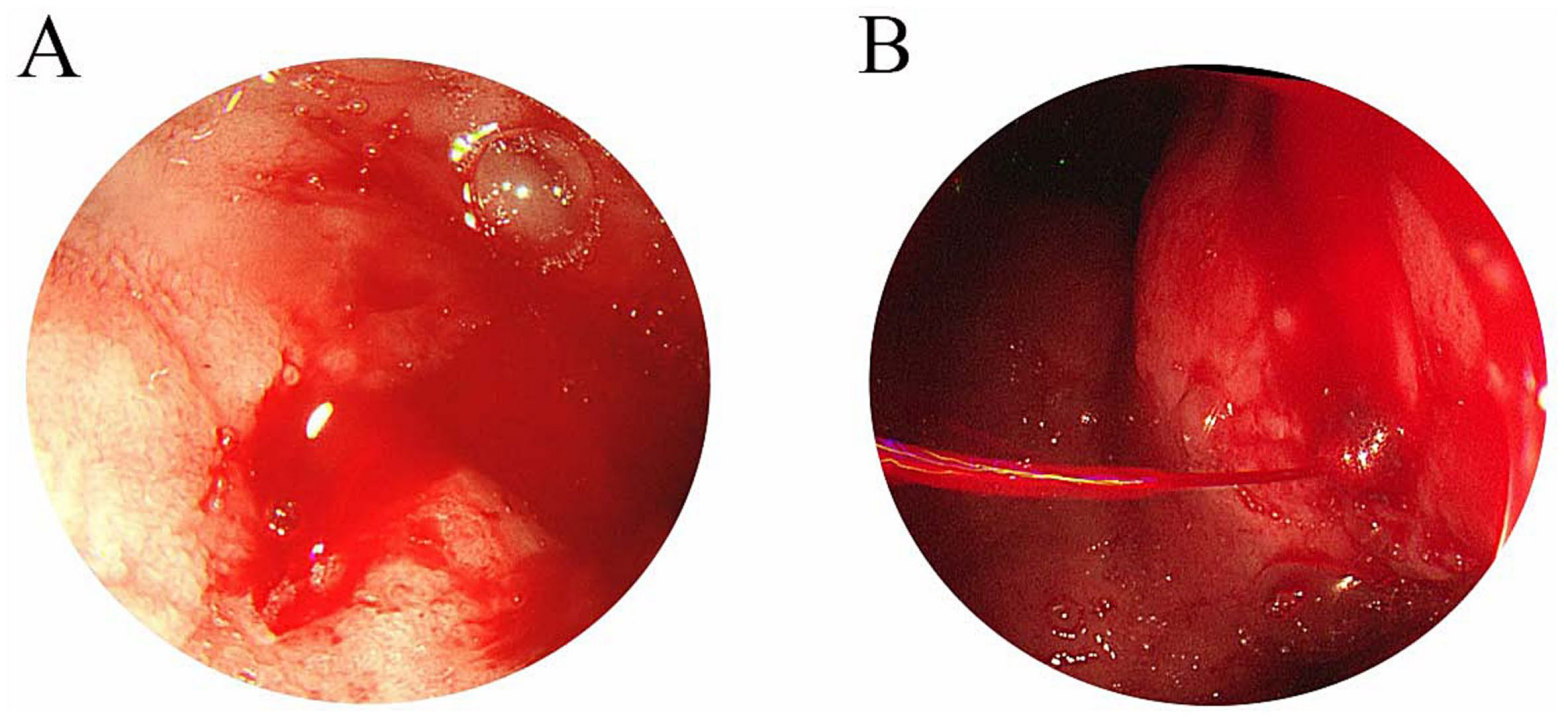
Endoscopic findings in UGIB. **(A)** Duodenal bulb ulcer with exposed vessel (Forrest IIa); **(B)** Gastric ulcer with active arterial spurring hemorrhage (Forrest Ia).

This review systematically synthesizes the role of the pyroptosis-immune-microbiome axis in UGIB pathogenesis and assesses its translational potential for risk prediction and personalized therapy. The manuscript is organized as follows: first, we summarize UGIB etiologies and risk factors; second, we detail the mechanisms of pyroptosis, immune dysregulation, and microbiome imbalance in UGIB; third, we analyze the pathological network underlying their interactions; fourth, we critically review established risk prediction models and biomarkers; finally, we evaluate conventional and emerging therapeutic strategies and outline future research directions (Figure 1). By systematically integrating advances in the pyroptosis-immunity-microbiome axis in acute upper gastrointestinal bleeding, this review categorizes evidence into three tiers: animal studies, *in vitro* mechanistic investigations, and clinical research, providing a critical appraisal of published findings. We examine methodological differences, inherent limitations, translational feasibility, and key challenges across study designs, defining the strength and clinical relevance of current evidence. This framework enhances the review’s scientific rigor and offers targeted guidance for future basic research and clinical translation in this field.

## 2 Etiology and risk factors of UGIB

### 2.1 Drug-related factors

Nonsteroidal anti-inflammatory drugs (NSAIDs) induce gastric mucosal injury primarily through selective inhibition of cyclooxygenase-1 (COX-1), which suppresses the biosynthesis of cytoprotective prostaglandins (notably PGE₂ and PGI₂). This dual disruption critically impairs the mucus-bicarbonate barrier and reduces mucosal blood flow, ultimately promoting peptic ulcer development and significantly elevating the risk of upper gastrointestinal bleeding (4). Antiplatelet drugs, warfarin, and novel oral anticoagulants (NOACs) have also been confirmed to significantly increase the incidence of UGIB (5). A nationwide Dutch cohort study revealed that despite declining prescription volumes of NSAIDs, the incidence of UGIB did not significantly decrease, suggesting that over-the-counter (OTC) drugs and concomitant medication use remain predominant risk factors (6). Proton pump inhibitors (PPIs) are a conventional measure for preventing drug-associated bleeding; however, long-term or high-dose usage may contribute to small intestinal microbiome dysbiosis and an elevated risk of lower gastrointestinal bleeding (7).

### 2.2 Infection and ulcers

*Helicobacter pylori* infection is a major contributing factor to peptic ulcer disease and associated bleeding; eradication therapy significantly reduces the incidence of rebleeding (2). *Helicobacter pylori* exacerbates gastric mucosal injury by activating inflammasomes, promoting pyroptosis, and inducing inflammatory cytokine release, and is closely associated with alterations in the composition of the gastric mucosal microbiome (3).

### 2.3 Age and genetic factors

Advanced age is an independent risk factor for UGIB. Elderly patients frequently present with multiple comorbidities (e.g., liver cirrhosis, renal impairment, and diabetes mellitus) and long-term polypharmacy, resulting in a significantly elevated risk of bleeding (5, 8). In patients with osteoarthritis, the majority exhibit overlapping exposure to risk factors such as NSAIDs and aspirin (9). Patients with cardiovascular disease receiving dual antiplatelet or anticoagulant therapy also necessitate close monitoring of bleeding risk (10). Genetic polymorphisms influence drug metabolism and mucosal vulnerability; for example, the CYP2C19 genotype determines PPIs metabolism rate, thereby affecting their preventive efficacy (11). PTGS1 and NOS3 genetic variants, among others, confer an increased risk of UGIB (12). Environmental factors such as smoking, alcohol abuse, high-salt/high-fat diet, and psychological stress compromise the mucosal barrier and alter microbiota composition, thereby indirectly elevating the incidence of bleeding (8).

## 3 Role of pyroptosis in gastric mucosal injury

Pyroptosis is an inflammasome-mediated mode of programmed cell death, wherein caspase-1/4/5/11 cleave gasdermin D (GSDMD) to form membrane pores, resulting in cellular rupture and the release of inflammatory mediators such as IL-1β and IL-18. Excessive pyroptosis causes tissue damage and propagates inflammation (8).

### 3.1 Pathogenic mechanism of GKN2-NLRP3 axis

Animal studies have demonstrated that upregulation of gastrokine 2 (GKN2) acts as a critical upstream activator of the NLRP3 inflammasome, inducing gastric epithelial pyroptosis via neutrophil recruitment and inflammatory cascade amplification (13). Suppression of GKN2 expression or administration of NLRP3 inhibitors (e.g., MCC950) reduces IL-1β release and neutrophil infiltration, thereby alleviating mucosal injury (14). This work first systematically elucidated the pathogenic role of pyroptosis in stress-induced gastric injury, providing a theoretical foundation for developing pyroptosis-targeted therapies (14).

### 3.2 Dual effects and targeted inhibition of pyroptosis

**Figure 2:**
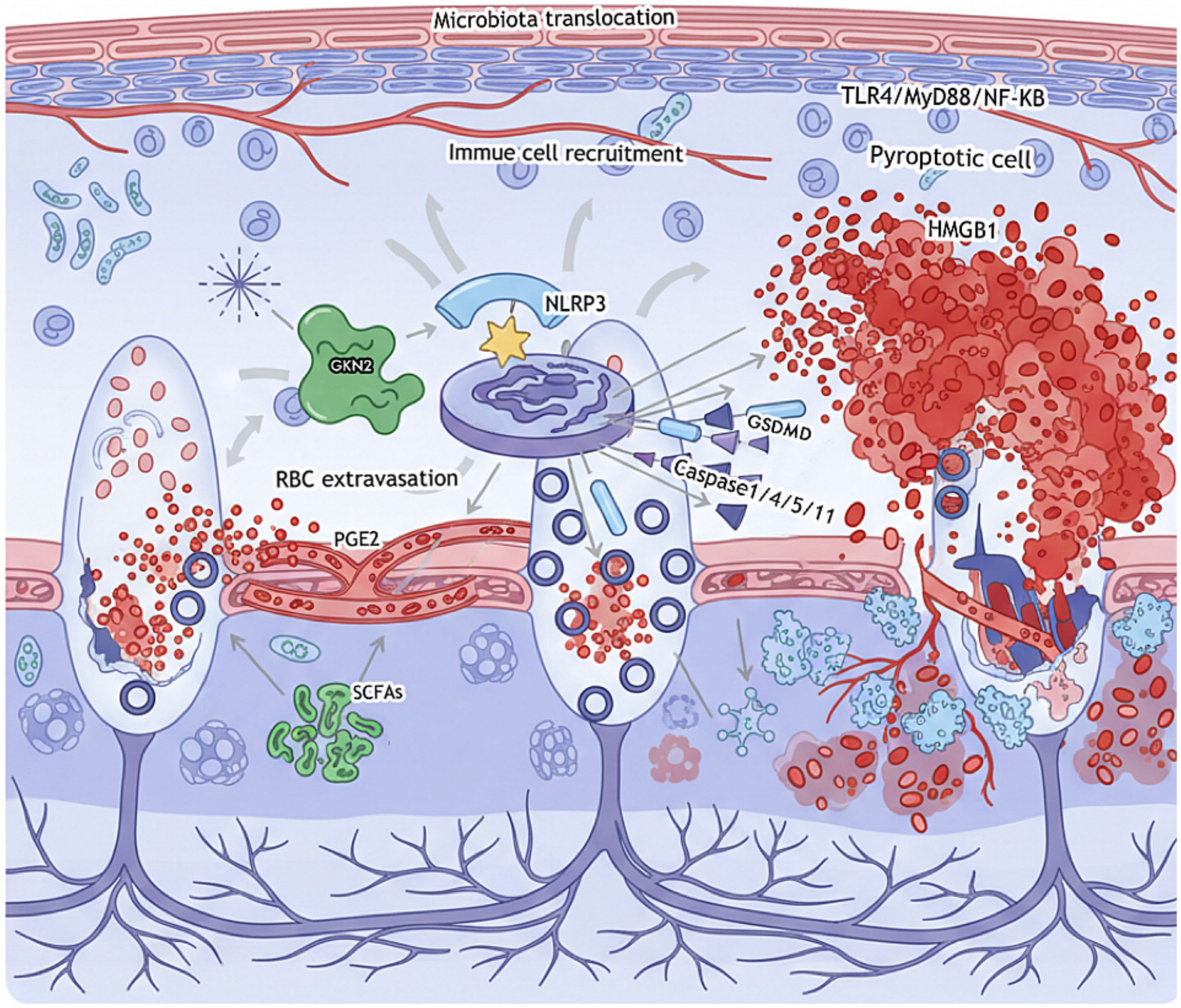
Pyroptosis-immunity-microbiota axis in upper gastrointestinal bleeding. This illustration depicts the pyroptosis-immunity-microbiota axis in upper GI bleeding. Damaged mucosal barrier allows microbiota translocation, which activates the TLR4/MyD88/NF-κB pathway and induces NLRP3 inflammasome assembly in gastric epithelial cells. Inflammasome-associated caspases (1/4/5/11) cleave GSDMD, causing pyroptotic cell lysis and release of HMGB1 and pro-inflammatory cytokines, which recruit immune cells and amplify inflammation. Vascular disruption leads to red blood cell (RBC) extravasation. Protective mediators including PGE₂ and SCFAs support mucosal healing and immune regulation to counteract pathological progression.

Pyroptosis induces cellular rupture and releases inflammatory cytokines, propagating inflammation and damaging adjacent tissues. However, pyroptosis activation, particularly via the NLRP3 inflammasome pathway, also upregulates cyclooxygenase-2 (COX-2) expression, driving the synthesis and release of prostaglandin E₂ (PGE₂), which promotes mucosal tissue repair and reflects a double-edged-sword effect (15, 16). Multiple natural compounds (e.g., fucoidan, C-phycocyanin, Phellinus linteus polysaccharides) and pharmaceuticals (e.g., rabeprazole, saxagliptin, ALDH2 activators, irbesartan) inhibit NLRP3 inflammasome activation, reduce IL-1β/IL-18 release, and alleviate gastric mucosal injury in animal experiments (17). However, these pyroptosis inhibitors lack validation in clinical trials, and excessive pyroptosis inhibition may compromise anti-infective defenses and tissue repair (Figure 2).

### 3.3 Critical appraisal and translational relevance

#### 3.3.1 Core findings by study type

(1) *Animal models*: Most evidence is derived from rodent models of stress-induced or ethanol-induced gastric injury. These studies consistently demonstrate that the GKN2-NLRP3 inflammasome axis drives gastric epithelial pyroptosis, and inhibition of GKN2/NLRP3 alleviates mucosal damage and reduces inflammatory cytokine release.
(2) *In vitro experiments*: Previous studies have confirmed that NLRP3 inflammasome activation and GSDMD cleavage are core events in pyroptosis in human gastric epithelial cell lines, and multiple compounds, including rabeprazole and fucoidan, can effectively suppress pyroptosis in a cell-autonomous manner.
(3) *Clinical evidence*: No direct clinical trials or cohort studies have validated pyroptosis pathways in human UGIB. Pyroptosis-related proteins (NLRP3, GSDMD, caspase-1) are only proposed as potential biomarkers based on extrapolation from gastric cancer and chronic gastritis studies.

#### 3.3.2 Methodological limitations

(1) *Animal models*: Rodent gastric mucosal structure, microbial composition, and inflammatory cascades differ markedly from those of humans, and most single-hit injury models based on stress or ethanol cannot recapitulate the complex etiology of clinical UGIB, such as drug-induced injury, cirrhosis, and advanced age with comorbidity.
(2) *In vitro experiments*: Monolayer cell cultures lack the three-dimensional mucosal microenvironment, immune cell infiltration, and microbiome crosstalk, which lead to over-simplified mechanistic conclusions.
(3) *Clinical translation*: No standardized detection methods for pyroptosis-related proteins in human gastric mucosa or peripheral blood, and lack of prospective clinical validation.

#### 3.3.3 Translational barriers to human UGIB

(1) Pyroptosis inhibitors (MCC950, AC-YVAD-CMK) have only been tested in preclinical animal models, with no phase I/II clinical trials for UGIB.
(2) Excessive inhibition of pyroptosis may impair host anti-infective defense and tissue repair, posing unknown safety risks in acute bleeding patients.
(3) No clear clinical cutoff values or detection protocols for pyroptosis biomarkers in risk stratification of UGIB.

## 4 Immune response and UGIB

### 4.1 Mucosal immunity and inflammatory markers

The gastric mucosal immune system serves as the first line of defense against pathogen invasion and for the maintenance of mucosal homeostasis (18). Upon mucosal barrier disruption or presence of bacterial toxins, dendritic cells and macrophages recognize pathogens via Toll-like receptors (TLRs), activating the NLRP3 inflammasome and triggering pyroptosis with consequent inflammatory amplification. Clinically utilized inflammatory markers include the NLR, C-reactive protein (CRP), and platelet-to-lymphocyte ratio (PLR). Multiple studies demonstrate that elevated NLR significantly associates with increased rebleeding rates, transfusion requirements, and mortality in UGIB patients (19, 20). Elevated CRP represents an independent risk factor for 30-day mortality in patients with non-variceal bleeding (21). Integrating NLR and CRP with conventional risk scoring systems significantly enhances predictive performance (22).

### 4.2 Autoimmunity and immune-mediated bleeding

In autoimmune gastritis, T cell-mediated attack and anti-parietal cell antibodies destroy parietal cells, inducing gastric mucosal atrophy and hemorrhagic tendency. IFN-*γ* and IL-17 secreted by CD4^+^ Th1/Th17 cells induce pyroptosis via activation of the ROS-NLRP3 pathway, exacerbating inflammatory responses. Clinically, corticosteroids and other immunosuppressants temporarily alleviate immune-mediated inflammation but necessitate vigilance for infection risk and progression of mucosal atrophy (23).

### 4.3 Critical appraisal and translational relevance

#### 4.3.1 Core findings by study type

(1) *Animal models*: Animal studies showed that mucosal immune disruption, TLR4 signaling, and Th1/Th17 cell polarization promoted gastric inflammation and pyroptosis, exacerbating mucosal injury.
(2) *In vitro experiments*: Immune cell (macrophage, dendritic cell) assays confirmed that inflammatory factors (IL-6, TNF-*α*) activated NLRP3 inflammasome and amplified pyroptosis in a positive feedback loop.
(3) *Clinical evidence*: Clinical studies consistently show that elevated NLR and CRP are independent risk factors for rebleeding, transfusion requirements, and 30-day mortality in UGIB patients, and their combination with traditional scoring systems may improve predictive accuracy.

#### 4.3.2 Methodological limitations

(1) *Animal models*: Rodent immune system development and cytokine profiles differ from humans, which cannot fully simulate the immune status of elderly or comorbid UGIB patients.
(2) *In vitro experiments*: Isolated immune cell cultures lack the mucosal barrier and systemic inflammatory microenvironment, failing to reflect *in vivo* immune crosstalk.
(3) *Clinical studies*: Most evidence comes from retrospective cohorts with potential selection bias, and few prospective multicenter studies have validated inflammatory marker cutoffs.

#### 4.3.3 Translational barriers to human UGIB

(1) No unified clinical cutoff values for NLR, CRP, and PLR in UGIB risk stratification, leading to inconsistent clinical application.
(2) Immunosuppressive agents (e.g., corticosteroids) used in immune-mediated gastritis carry infection risks, with no clear dosage and duration guidelines for UGIB.
(3) Immune-related therapeutic targets lack prospective clinical trials for UGIB.

## 5 Microbiome dysbiosis and gastric mucosal pathology

### 5.1 Causes and consequences of dysbiosis

The gastrointestinal microbiota plays a crucial role in maintaining mucosal barrier integrity, mediating nutrient digestion, and regulating immune tolerance (22). Multiple factors, including dietary patterns, psychological stress, antibiotic exposure, NSAID and PPI use, and infections, can induce microbiota dysbiosis. Dysbiosis is characterized by reduced microbial diversity, depletion of beneficial probiotics, and overgrowth of opportunistic pathobionts, which compromise mucosal barrier integrity (23), permit translocation of pro-inflammatory substances, and trigger inflammasome activation. Mendelian randomization studies suggest that microbiota dysbiosis promotes peptic ulcer development through reduced SCFA production (24). Small-intestinal microbiota dysbiosis in long-term PPI users is associated with an elevated bleeding risk. Systematic reviews and meta-analyses confirm that PPI therapy significantly increases the risk of lower gastrointestinal bleeding (OR = 1.54, 95% CI: 1.32–1.80), particularly small-bowel bleeding, with greater risk in patients concurrently using aspirin or NSAIDs (25, 26).

### 5.2 Interaction among microbiome, pyroptosis, and immunity

**Figure 3:**
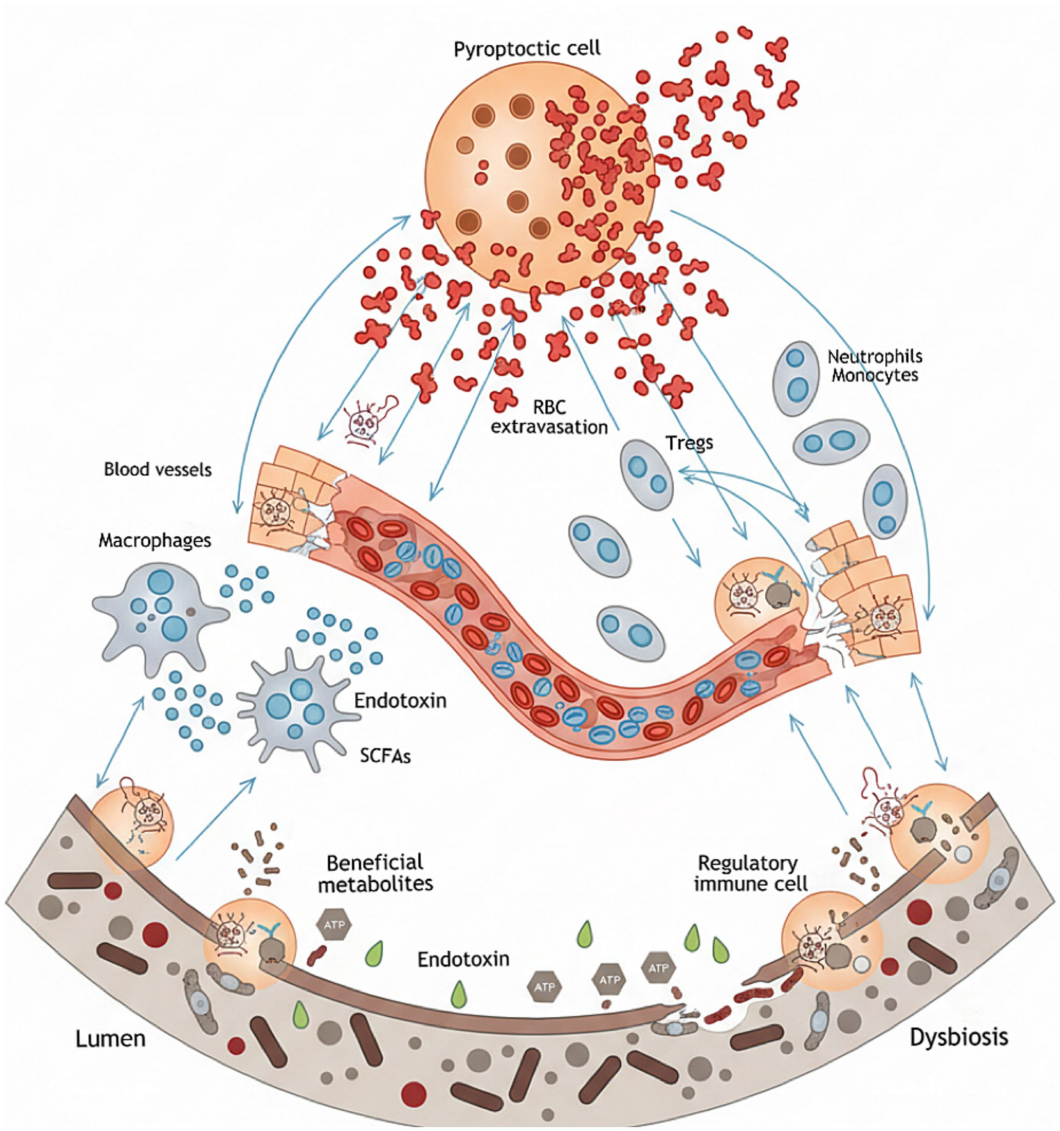
Gut dysbiosis induces barrier disruption and pyroptosis-driven inflammation. Gut dysbiosis elevates endotoxin, depletes SCFAs/beneficial metabolites, and impairs Treg function, activating macrophages. RBC extravasation and pyroptosis release inflammatory mediators, recruiting immune cells and driving vascular inflammation.

The microbial metabolite butyrate exerts well-documented anti-inflammatory and inflammasome-inhibitory effects via HDAC suppression and autophagy enhancement (27, 28). Although no direct clinical studies in UGIB patients are currently available, in clinically relevant settings such as peptic ulcer disease, stress-induced gastric mucosal injury, and long-term PPI use-related intestinal dysbiosis, significant reductions in intestinal butyrate levels have been documented, and butyrate-deficiency-associated dysbiosis is a recognized risk factor for UGIB (29, 30). Conversely, bacterial endotoxins (e.g., lipopolysaccharide) and extracellular ATP activate complementary pro-inflammatory pathways—LPS engages TLR4 signaling to induce NF-κB-dependent pro-inflammatory cytokine transcription, whereas ATP activates the P2X7 receptor to trigger NLRP3 inflammasome assembly, culminating in gasdermin D-mediated pyroptosis and IL-1β release (31, 32). Consequently, microbiota dysbiosis disrupts mucosal barrier function through downregulation of tight junction proteins and reduced antimicrobial peptide production, thereby increasing susceptibility to mucosal injury (33). Importantly, this relationship is bidirectional: host inflammatory responses also remodel gut microbial ecology—IL-1β and IL-18 induce antimicrobial peptides that preferentially deplete obligate anaerobes, whereas epithelial injury compromises the anaerobic luminal environment and facilitates the expansion of facultative pathobionts such as Enterobacteriaceae (34). This self-perpetuating cycle of dysbiosis and inflammation creates a vulnerable mucosal environment that may contribute to the pathogenesis and impaired healing of gastrointestinal ulcers and bleeding complications in susceptible individuals, although direct causal evidence in human UGIB still requires further validation (Figure 3).

### 5.3 Critical appraisal and translational relevance

#### 5.3.1 Core findings by study type

(1) *Animal models*: Germ-free or antibiotic-treated mouse models confirm that gut microbiome dysbiosis reduces SCFA production, impairs mucosal barrier, and activates TLR4/NF-κB pathway to promote pyroptosis and gastric injury.
(2) *In vitro experiments*: *In vitro* experiments indicate that LPS and microbial metabolites directly activate inflammasomes and disrupt tight junction proteins in gastric epithelial cells.
(3) *Clinical evidence*: Clinical studies demonstrate that long-term PPI usage induces small intestinal dysbiosis and increases lower GI bleeding risk. Dysbiosis is associated with peptic ulcer and UGIB by reducing SCFA levels.

#### 5.3.2 Methodological limitations

(1) *Animal models*: Murine gut microbiome composition is drastically different from humans. The results acquired from germ-free or antibiotic-intervened models cannot fully represent clinical dysbiosis.
(2) *In vitro experiments*: The lack of a complex microbial community and host metabolic microenvironment makes it difficult to reproduce microbiome-immune crosstalk *in vivo*.
(3) *Clinical studies*: No standardized protocols exist for microbiome sampling, sequencing, or bioinformatic analysis, and small sample sizes and single-center designs limit generalizability.

#### 5.3.3 Translational barriers to human UGIB

(1) No consensus on microbiome signatures or metabolite biomarkers for UGIB risk prediction and diagnosis.
(2) Probiotics, prebiotics, and FMT lack standardized strains, dosages, and treatment courses for UGIB, and clinical efficacy is highly heterogeneous.
(3) Long-term PPI-induced dysbiosis requires balanced risk–benefit assessment, with no clear intervention strategies for clinical practice.

## 6 The interacting network of Pyroptosis-immunity-microbiome

### 6.1 Pyroptosis mediators disrupt epithelial barrier and enhance inflammation

During pyroptosis, cleavage of the GSDMD perforates the cell membrane, resulting in massive extrusion of cellular contents (e.g., IL-1β, IL-18) (35). These inflammatory mediators induce vasodilation, promote leukocyte migration, and drive the recruitment and activation of neutrophils, macrophages, and T cells (14). Pyroptosis is associated with the downregulation of epithelial tight junction proteins, thereby compromising the mucosal barrier. Additionally, the release of IL-1β, IL-18, HMGB1, and other pro-inflammatory mediators upon cell rupture attracts dendritic cells and macrophages, thereby enhancing antigen presentation. The disruption of the mucosal barrier and the infiltration of immune cells facilitate the translocation of gut microbiota or pathogens, providing a novel stimulus for subsequent inflammatory responses (35).

### 6.2 Immune cell factors promote pyroptosis and amplify inflammation

Various inflammatory factors, such as IL-6 and TNF-*α*, can promote pyroptosis through the NLRP3 inflammasome or caspase pathways. Studies have shown that IL-6 can activate the NLRP3 inflammasome and induce the secretion of IL-1β/IL-18 in oral squamous cell carcinoma. Silencing NLRP3 inhibits IL-6-induced pyroptosis (36). Caspase-3, activated by TNF-*α* or chemotherapeutic agents, cleaves the gasdermin E (GSDME) protein, generating the GSDME-N fragment. This fragment not only induces cell membrane pore formation but also activates the inflammasome, promoting further release of IL-1β and IL-18 (37). These immune mediators facilitate the activation of pyroptosis, and the subsequent release of cytokines such as IL-1β and IL-18 further stimulates immune cells to secrete additional pro-inflammatory cytokines, thereby creating a positive feedback loop that amplifies the inflammatory response.

### 6.3 Microbial dysbiosis activates pyroptosis via the TLR4/MyD88/NF-κB pathway

The lipopolysaccharide (LPS) and endotoxins generated by microbial dysbiosis can bind to TLR4, activating the MyD88/NF-κB signaling pathway, which induces NLRP3 inflammasome assembly and IL-1β release (38). The study demonstrated that LPS-TLR4 signaling upregulates IL-1 receptors, thereby enhancing macrophage sensitivity to IL-1β, which ultimately triggers caspase-1-dependent pyroptosis. Inhibition of TLR4 or MyD88 significantly attenuates this pyroptotic response (38). Following pyroptosis-induced disruption of the mucosal barrier, intestinal microbiota translocation occurs, further stimulating the TLR4/NF-κB pathway. On the other hand, SCFAs produced by healthy microbiota promote the generation of regulatory T cells (Tregs). Antibiotic treatment or germ-free mice lacking SCFAs exhibit a reduction in peripheral Treg numbers, while butyrate supplementation can restore Treg cell populations (39). Microbial dysbiosis and the resulting deficiency in SCFAs lead to impaired immune tolerance, making the system more prone to inflammatory responses. This, in turn, exacerbates pyroptosis and mucosal damage. These studies indicate that pyroptosis, immune responses, and microbial dysbiosis form a self-amplifying network: mediators released during pyroptosis recruit inflammatory cells and disrupt the barrier, while immune cell factors further activate inflammasomes and pyroptosis. Dysbiotic microbiota, through LPS and other stimuli, activate the TLR4/MyD88 signaling pathway and reduce SCFAs, leading to sustained amplification of pyroptosis and inflammation. Therefore, combined interventions targeting pyroptosis, immune modulation, and microbiome restoration may be more effective than single-target strategies in treatment.

### 6.4 Integrated conceptual framework: bidirectional interactions and feedback loops

Pyroptosis, immune responses, and gut microbiome do not function independently, but form a bidirectionally regulated and self-amplifying closed-loop pathogenic axis that collectively drives the occurrence and progression of UGIB.

(1) Forward regulation from microbiome to pyroptosis and immunity.

Gut microbiome dysbiosis activates the TLR4/MyD88/NF-κB pathway through LPS, directly inducing NLRP3 inflammasome assembly and pyroptosis activation. Meanwhile, the reduction of microbial metabolite SCFA impairs immune tolerance, promotes pro-inflammatory immune phenotype polarization, and further amplifies the inflammatory response.

(2) Reverse regulation from pyroptosis to immunity and microbiome.

During pyroptosis, GSDMD membrane perforation causes cell lysis and releases pro-inflammatory mediators such as IL-1β, IL-18 and HMGB1, which recruit the infiltration of neutrophils, macrophages and other immune cells and activate systemic inflammation. The inflammatory microenvironment disrupts the intestinal anaerobic environment, leading to the depletion of beneficial bacteria and overgrowth of opportunistic pathogens, thus aggravating microbiome dysbiosis.

(3) Positive feedback from immunity to pyroptosis and microbiome.

Inflammatory factors such as IL-6 and TNF-*α* secreted by immune cells further induce pyroptosis by activating the NLRP3 inflammasome/caspase pathway. Meanwhile, inflammation-mediated mucosal barrier injury causes microbial translocation, which continuously activates inflammatory and pyroptotic pathways.

(4) Self-amplifying pathogenic closed loop.

**Figure 4:**
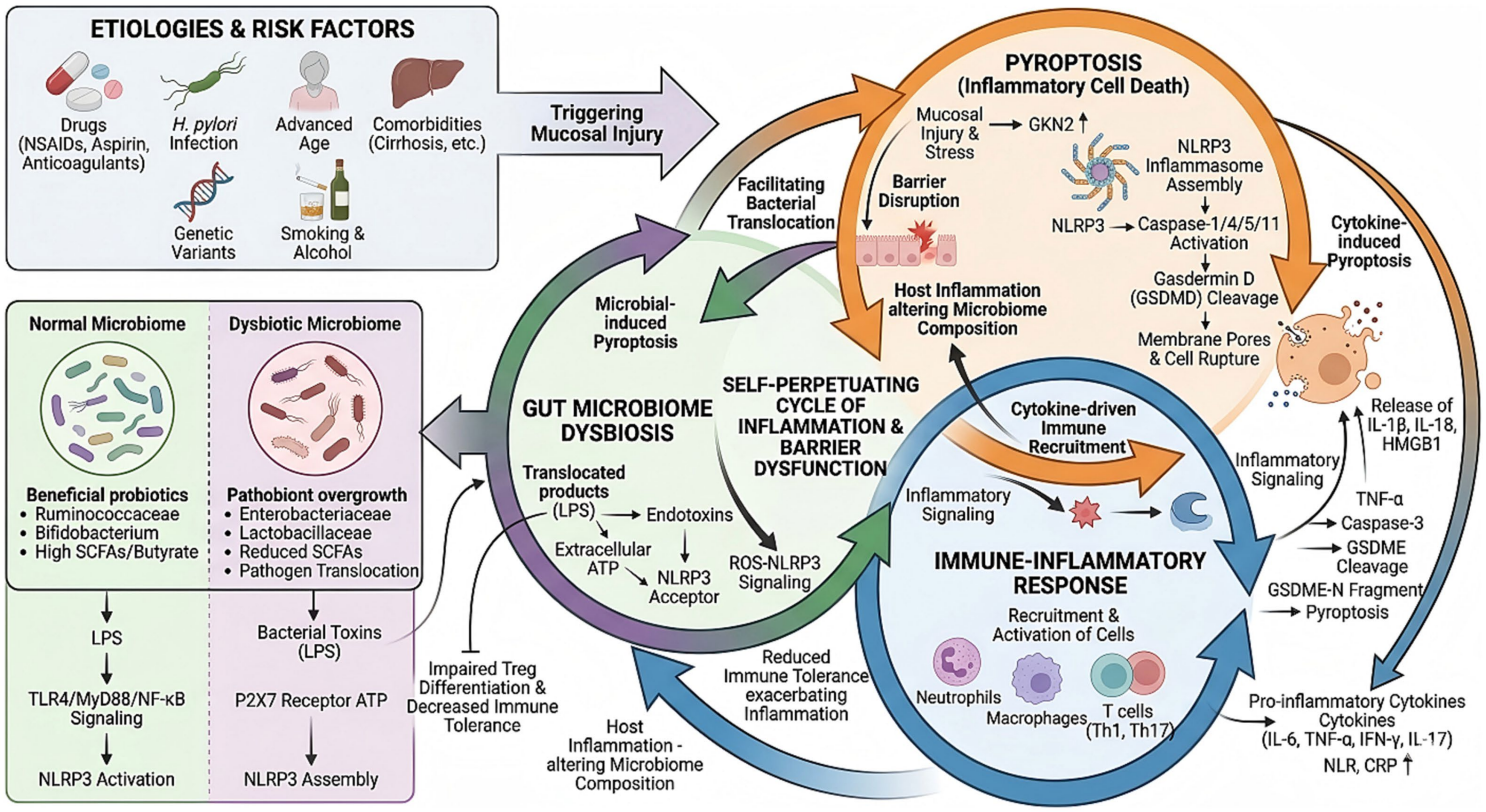
Bidirectional interactions and self-amplifying feedback loops for pyroptosis-immunity-microbiome axis in UGIB. This figure illustrates the core pathogenesis of UGIB: Risk factors including drugs and *Helicobacter pylori inf*ection trigger initial gastric mucosal injury, activating NLRP3 inflammasome-mediated pyroptosis. Pro-inflammatory cytokines released by pyroptotic cells recruit immune cells, forming a positive feedback loop between pyroptosis and immunity. Meanwhile, gut microbiota dysbiosis (reduced beneficial bacteria, expanded pathobionts) continuously activates inflammation and pyroptosis via endotoxins. Coupled with bacterial translocation from disrupted mucosal barriers, this ultimately forms a self-amplifying pathological vicious cycle that drives the onset and progression of UGIB.

The three components form an irreversible positive feedback loop: microbiome dysbiosis, pyroptosis activation, immune amplification, further microbiome dysbiosis, mucosal barrier disruption, vascular injury, and bleeding, which ultimately constitute the core pathological basis of UGIB. This integrated framework clarifies the bidirectional interactions of the pyroptosis-immunity-microbiome axis, breaks through the limitations of single-factor research, and provides a core theoretical basis for multi-target combined intervention of UGIB (Figure 4).

## 7 Risk prediction models and biomarkers

### 7.1 Traditional risk scores

Glasgow-Blatchford Score (GBS): The GBS was originally developed in 2000 using data from 1,748 patients, with the aim of predicting whether patients would require blood transfusion, endoscopic treatment, or surgical intervention (40). Multinational studies have validated its effectiveness and concluded that patients with a GBS of 0–1 do not require hospitalization and can be safely managed on an outpatient basis (40). A multicenter study demonstrated that the GBS exhibits superior performance in predicting the need for blood transfusion. However, it is less effective than the Rockall score in forecasting mortality or the requirement for medical intervention (41). Rockall Score: The Rockall score was developed in 1996 based on a cohort of 4,185 patients with UGIB to predict mortality risk (40). The complete score comprises five variables: age, shock status, comorbidities, endoscopic diagnosis, and stigmata of recent hemorrhage; the first two are clinical parameters, while the latter three require endoscopic assessment. The Rockall score demonstrates superior predictive capability for mortality compared with the GBS (41), but requires endoscopic findings and therefore cannot be calculated immediately upon hospital admission. AIMS65 score: The AIMS65 score was developed in the United States in 2011 using a derivation cohort of 29,222 patients and validated in an independent cohort of 32,504 patients. This scoring system is simple and memorable, comprising only five objective parameters: albumin level, international normalized ratio (INR), altered mental status, systolic blood pressure, and age ≥65 years (40, 42). It is primarily utilized to predict in-hospital mortality and to assess length of hospital stay and healthcare costs. Research demonstrates that AIMS65 exhibits a high AUROC for predicting in-hospital mortality and outperforms the Rockall score in this domain (40). However, AIMS65 demonstrates inferior performance in predicting rebleeding and clinical intervention requirements. A multicenter comparative analysis revealed that the PNED score (incorporating rebleeding-specific variables) achieved an AUROC of 0.85, whereas GBS, Rockall score, pRS, and AIMS65 yielded AUROCs of only 0.62, 0.64, 0.62, and 0.60, respectively (40).

In summary, GBS, the Rockall score, and AIMS65 each have distinct clinical roles: GBS is ideal for rapid early assessment without endoscopy and identifying low-risk patients; the Rockall score is suited for evaluating mortality and rebleeding risk after incorporating endoscopic findings; and AIMS65 provides a simple, objective calculation that effectively predicts in-hospital mortality but has limited utility for predicting rebleeding and therapeutic needs. Consequently, clinical practice often combines multiple scoring systems for comprehensive risk stratification (Table 1).

**Table 1:** Comparison of risk scoring systems for UGIB.

Scoring system	Core parameters	Primary purpose
GBS	Hb, BUN, SBP and pulse, comorbidities, symptoms (melena, syncope)	Assess need for transfusion/endoscopic therapy/surgery; early risk stratification
Rockall score	Age, shock index, comorbidities, endoscopy findings	Estimate rebleeding and mortality risk; post‑endoscopy prognosis
AIMS65	Albumin, INR, mental status, low SBP, age ≥65	Predict in-hospital mortality, length of stay, and healthcare costs

### 7.2 Emerging biomarkers and multi-omics integration

#### 7.2.1 Applications and advantages of inflammatory marker models

Multiple studies have incorporated inflammatory markers—such as NLR, CRP, CLR, PLR, and D-dimer—into UGIB risk prediction models to enhance conventional scoring systems’ accuracy. Using community emergency department data, researchers integrated NLR with GBS and Rockall score to develop NLR-GBS and NLR-Rockall models. The NLR-Rockall demonstrated a marginally higher AUC for predicting in-hospital mortality (0.763) versus Rockall alone (0.747), while NLR-GBS showed superior performance for composite adverse outcomes (AUC 0.687 vs. 0.662 for GBS), confirming inflammatory markers augment traditional scores (19). Another clinical study constructed a predictive model using *H. pylori* infection status, ulcer stage, NSAID use, NLR, and CLR, achieving an AUC of 0.921—significantly higher than any single parameter—with excellent model fit (22). D-dimer has also been validated for rebleeding prediction. A 2023 study developed a novel model incorporating blood lactate, neutrophil percentage, platelet count, albumin, and D-dimer, yielding an AUC of 0.746 for rebleeding prediction, substantially outperforming AIMS65 (AUC 0.619). This superiority persisted in both training and validation cohorts by C-index analysis (43). Multivariable regression identified blood lactate, neutrophil percentage, and D-dimer as rebleeding risk factors, whereas platelet count and albumin exhibited protective associations (44).

#### 7.2.2 Apoptosis-related proteins and gene polymorphisms as potential biomarkers

Pyroptosis-related proteins—including NLRP3 inflammasome components, the pore-forming protein GSDMD, and caspases-1/3/8—play critical roles in inflammation and gastric mucosal injury, and are regarded as potential novel biomarkers. Multi-omics analysis of gastric cancer specimens revealed frequent amplification of pore-forming and inflammasome genes (GSDMA, GSDMB, GSDMC, GSDMD, and NLRP3) in tumor tissues, with significantly elevated mRNA expression compared to normal mucosa (45). These pyroptosis-related genes show enrichment in multiple inflammatory pathways (NOD-like receptor signaling, TNF, and IL-17 signaling) (45). Additionally, analysis of metabolomic profiling data revealed that the expression patterns of 33 pyroptosis-related genes completely distinguished gastric cancer patients from normal controls, demonstrating their diagnostic potential (45). However, clinical studies on UGIB patients remain limited, and standardized clinical assays for apoptosis-related proteins have not yet been established. Large-scale studies are needed to validate their predictive value.

#### 7.2.3 Microbiome multi-omics integration and future perspectives in machine learning

The gut microbiota is closely associated with pyroptosis and inflammation, but utilizing microbial signatures for UGIB risk assessment remains in exploratory phases. During gastrointestinal mucosal injury, microbiota dysbiosis and endotoxin release activate the TLR4-MyD88-NF-κB pathway, inducing inflammatory cytokines such as IL-1β and pyroptosis (38). Consequently, microbiome signatures and their metabolites may serve as future risk biomarkers, warranting in-depth exploration using metagenomic and metatranscriptomic technologies. With advances in high-throughput sequencing and big data analytics, integrating multi-omics information—whole-genome sequencing, transcriptomics, metabolomics, and gut microbiome data—with machine learning algorithms enables development of individualized predictive models. Recent machine learning studies demonstrate that XGBoost and CatBoost models predict in-hospital mortality in UGIB patients with an AUC of 0.84, substantially outperforming GBS and Rockall score (AUC 0.68 and 0.62, respectively) (46). These algorithms process high-dimensional nonlinear data, providing the technical foundation for future multi-omics integration. Recent studies have preliminarily combined multiple inflammatory markers (e.g., NLR, CRP) via machine learning models, significantly enhancing risk assessment accuracy. This suggests that future frameworks integrating genetic polymorphisms, pyroptosis-related protein expression, and microbiome signatures through machine learning could establish more precise individualized predictive tools, facilitating early identification and intervention for high-risk UGIB patients.

## 8 Therapeutic strategies and emerging targets

### 8.1 Acute management and combined individualized therapy

Acute management of UGIB involves rapid hemodynamic stabilization and hemostatic measures. Endoscopic hemostasis is the primary intervention (47). Pharmacologically, high-dose PPIs should be administered early to suppress gastric acid secretion and stabilize clot formation (48). For esophageal variceal bleeding, somatostatin analogs (octreotide) or terlipressin combined with endoscopic therapy constitutes the standard regimen; short-term antibiotic prophylaxis reduces infection risk (49).

Preventive strategies encompass *H. pylori* eradication, prudent use of NSAIDs and antithrombotics, concomitant administration of mucoprotective agents, and lifestyle modifications (50). For patients on long-term aspirin or anticoagulant therapy, co-administration of mucoprotective agents or low-dose PPIs may be considered; however, potential effects on the small intestinal microbiota warrant careful risk–benefit evaluation (51). Given the complex interplay among pyroptosis, immunity, and microbiota, monotherapy targeting pyroptosis may be insufficient to improve prognostic outcomes. Combined use of pyroptosis inhibitors, anti-inflammatory agents, and probiotics, alongside early endoscopic and vascular interventions, may enhance therapeutic efficacy in high-risk patients. Integrating clinical features, inflammatory markers, and microbiome profiles to develop precision phenotyping-based individualized treatment protocols represents a promising future direction in clinical management (52).

### 8.2 Targeting pyroptosis and inflammasomes

In gastric mucosal injury (the core pathological basis of UGIB), NLRP3/caspase-mediated pyroptosis is a key driver. Animal experiments based on UGIB-related gastric injury models (stress, ethanol, or NSAIDs-induced) confirm that inflammasome inhibitors alleviate mucosal damage. For instance, the caspase-1-specific inhibitor AC-YVAD-CMK significantly suppresses pyroptosis and reduces inflammatory responses in mice with ethanol-induced acute gastric injury (14). Notably, most studies on MCC950 and other pyroptosis inhibitors are derived from non-UGIB models such as ulcerative colitis and acute pancreatitis, and direct evidence in human UGIB is still absent. Furthermore, researchers have identified that multiple pharmaceuticals and natural compounds—including rabeprazole, fucoidan, ALDH2 activators (e.g., Alda-1), the dipeptidyl peptidase-4 inhibitor saxagliptin, the natural protein C-phycocyanin, and the angiotensin II receptor blocker irbesartan—inhibit NLRP3 inflammasome activation and GSDMD-mediated pyroptosis, reduce IL-1β release, attenuate inflammatory responses, and promote gastric mucosal healing in animal models (14). As the most extensively studied selective NLRP3 inhibitor, MCC950 has demonstrated significant reduction of IL-1β and IL-18 levels and amelioration of NLRP3-mediated tissue damage across multiple inflammatory disease models; some studies further observed its capacity to modulate intestinal microbiota composition and attenuate inflammation (53). Although data from gastric mucosal injury models remain limited, these findings provide a theoretical foundation for applying NLRP3 inhibitors in gastroduodenal diseases.

### 8.3 Microbiome modulation strategies

Gut microbiota dysbiosis compromises mucosal barrier integrity and exacerbates inflammation in UGIB. Probiotic supplementation can repair barrier function through multiple mechanisms in UGIB-relevant gastric mucosal injury models, including peptic ulcer disease and NSAID-induced gastric damage (54, 55). Reviews indicate that probiotics not only stimulate epithelial renewal but also enhance tight junction protein expression and mucus production, promote antimicrobial peptide secretion, and cooperate with commensal bacteria to suppress pathobiont expansion (56). Probiotics such as Lactobacillus and Bifidobacterium upregulate tight junction proteins, including ZO-1, occludin, and claudins, thereby enhancing epithelial barrier integrity and reducing intestinal permeability (57). Prebiotics are defined as substrates selectively utilized by host microorganisms that increase short-chain fatty acid production by providing fermentable fiber; SCFAs serve as energy sources for the intestinal mucosa and exert anti-inflammatory effects. Clinical trials have shown that high-fiber diets enriched with oat bran or inulin (fructooligosaccharides) significantly increase fecal butyrate concentrations while reducing abdominal pain and inflammatory markers (58). Fecal microbiota transplantation (FMT) reconstructs a diverse gut microbiota by transferring fecal microbiota from healthy donors to patients. In a randomized controlled trial in ulcerative colitis, patients receiving FMT achieved a clinical remission rate of 24%, significantly higher than the 5% observed in the placebo group (59). A systematic review of 14 cohort studies and 4 randomized controlled trials showed that FMT achieved a clinical remission rate of 28% in active ulcerative colitis, compared with only 9% in control groups (57). High-fiber diets not only provide prebiotics that promote the proliferation of butyrate-producing bacteria such as *Faecalibacterium prausnitzii*, but also reduce the proportion of pathobionts, thereby facilitating restoration of the intestinal microenvironment. Consequently, judicious use of antibiotics and PPIs, adoption of high-fiber dietary patterns, and appropriate probiotic supplementation may help maintain gut microbial homeostasis, enhance mucosal barrier integrity, and modulate inflammatory responses.

## 9 Evidence strength, limitations, and translational prospects

### 9.1 Evidence hierarchy pyramid for the pyroptosis-immunity-microbiome Axis in UGIB

Current evidence can be structured into a “four-level evidence pyramid” based on clinical reliability in this field, ranked from highest to lowest as follows:

(1) Randomized controlled trials (RCTs).

Only small-sample RCTs exist for validating the predictive value of inflammatory markers (NLR, CRP) combined with traditional scores and probiotic adjuvant interventions. No UGIB-specific RCTs have been conducted for pyroptosis inhibitors or precise microbiome modulation.

(2) Clinical cohort/retrospective studies.

This is the core source of current clinical evidence, confirming that NLR and CRP can be used for UGIB risk stratification, long-term PPI usage induces dysbiosis and increases bleeding risk, and *Helicobacter pylori* infection is associated with UGIB. However, most studies are single-center with small sample sizes and lack unified standards.

(3) Experiments *in vitro.*

These studies clarify the molecular mechanisms of core pyroptosis pathways (GKN2-NLRP3-GSDMD), TLR4/NF-κB inflammatory pathway, and microbial metabolites (SCFA, LPS). Due to the absence of *in vivo* microenvironment and immune-microbiome crosstalk, these conclusions cannot be directly translated clinically.

(4) Animal model studies.

Most researches are derived from rodent gastric injury models, confirming that pyroptosis, immune disorders, and microbiome dysbiosis synergistically induce mucosal injury. The gastric mucosal structure, microbiome composition, and immune system of animals differ significantly from humans, resulting in poor extrapolation of results.

### 9.2 Common methodological limitations in this field

#### 9.2.1 Severe disconnection between basic research and clinical practice

Core mechanisms of pyroptosis and microbiome-immune crosstalk are only verified in animal/cell models, with no direct mechanistic evidence from human UGIB tissue samples, making basic findings unable to translate to clinical applications.

#### 9.2.2 Extreme lack of translational research

Novel targets such as pyroptosis inhibitors and microbiome modulation remain at the preclinical stage, with no Phase I/II clinical trials in UGIB patients and no translational application pathways.

#### 9.2.3 Insufficient quality of clinical research

There is a lack of large-sample, multicenter, prospective studies. The absence of standardized protocols for biomarker detection and microbiome sequencing results in high heterogeneity and poor reproducibility.

#### 9.2.4 Simplistic intervention strategies

Existing studies mostly focus on a single target (e.g., only inhibiting pyroptosis or only supplementing probiotics), without designing combined intervention strategies targeting the positive feedback loop of the pyroptosis-immunity-microbiome axis.

### 9.3 Priority translational research directions in the future

#### 9.3.1 Conduct multi-center clinical studies

Large-sample prospective cohorts should be established to verify the clinical value of pyroptosis-related proteins and microbiome markers in UGIB risk prediction and to develop unified detection standards and cutoff values.

#### 9.3.2 Promote clinical translation of novel targets

Conduct Phase I/II clinical trials of pyroptosis inhibitors and precise probiotics/prebiotics to evaluate their safety and efficacy in UGIB treatment and rebleeding prevention.

#### 9.3.3 Develop combined intervention strategies

Based on the crosstalk mechanism of the pyroptosis-immunity-microbiome axis, individualized combination regimens incorporating pyroptosis inhibition, immune regulation, and microbiome restoration should be developed.

#### 9.3.4 Integrate multi-omics and artificial intelligence

Integrating genomic, metabolomic, and microbiome data to construct a precise UGIB risk prediction model via machine learning may facilitate early screening and individualized management of high-risk populations.

#### 9.3.5 Optimize clinical medication regimens

To clarify the dysbiosis risk of long-term PPI use, establish a balanced medication strategy of “acid suppression + microbiome protection”, and reduce the risk of total gastrointestinal bleeding.

## 10 Controversies and conclusion

Multiple studies have demonstrated that PPIs effectively reduce upper gastrointestinal bleeding in high-risk patients (57). However, this protective effect does not extend to the lower gastrointestinal tract. Long-term PPI therapy may exacerbate small-bowel injury and increase the risk of lower gastrointestinal bleeding, potentially through acid-suppression-induced alterations in the intestinal microbiome (60). Observational data show that among patients on antiplatelet therapy, PPIs reduce gastroduodenal bleeding but do not reduce lower gastrointestinal bleeding and are associated with higher rates of small-bowel injury. In gastric mucosal injury models, inhibitors targeting pyroptosis, especially the NLRP3 inflammasome and caspase-1/11, show pronounced protective effects. Pyroptosis is a double-edged sword: moderate pyroptosis helps clear pathogens and initiate immune responses, whereas overactivation disrupts the mucosal barrier and exacerbates inflammation (61). Nevertheless, the clinical safety of pyroptosis inhibitors remains uncertain because many agents lack specificity, may inhibit off-target caspases, and could impair host defense (31). Probiotics, prebiotics, and FMT can restore gut microbial homeostasis and reinforce barrier function, but heterogeneity in strains, doses, and study designs prevents firm conclusions (62). Intervention during acute bleeding episodes also requires caution because live microbial preparations may increase the risk of bacteremia or other complications in severely ill or immunocompromised patients (63). Precision microbiome-based therapies therefore require rigorous patient stratification, strict safety monitoring, and ethical oversight (64). In the future, integrated multi-omics combined with artificial intelligence may help identify high-risk populations and support personalized intervention strategies (65).

UGIB is an acute condition resulting from multiple interacting factors, with pathological mechanisms involving diverse pathways such as pyroptosis, immune responses, and microbiome dysbiosis. Key risk factors include drug use, *H. pylori* infection, age, and comorbidities (66). Pyroptosis, driven by inflammasomes, causes cell lysis and releases pro-inflammatory mediators like IL-1β and IL-18, which exacerbate mucosal inflammation yet may also facilitate repair. Immune responses affect bleeding risk via autoimmunity and amplification of inflammation. Microbiome dysbiosis compromises the mucosal barrier and influences inflammasome activation. Systemic inflammatory markers (e.g., NLR, CRP) alongside traditional risk scores are valuable for predicting rebleeding and mortality. Emerging therapies targeting pyroptosis and modulating the microbiota show promise, though further clinical validation is needed.

Future efforts should prioritize multicenter, large-sample randomized controlled trials to validate the efficacy and safety of pyroptosis inhibitors and microbiome modulation approaches. Additionally, leveraging multi-omics data and machine learning techniques to develop personalized risk prediction models could enable precise prevention and treatment, ultimately reducing the incidence and mortality of UGIB.
